# Supplementary material for: It Runs in the Bromodomain Family: Speckled Proteins (SP) Play a Role in the Antitumor Immune Response in Solid Tumors
Source: Int J Mol Sci. 2022 Dec 29;24(1):549. doi: 10.3390/ijms24010549 (PMC9820261; doi:10.3390/ijms24010549)
Supplement: Supplementary file 1 [file ijms-24-00549-s001.zip › ijms-2039073-supplementary.pdf]

It runs in the Bromodomain family: Speckled proteins (SP) play a role in anti-tumor immune response in solid tumors

Monika Anna Rosochowicz, Julia Maria Lipowicz, Marianna Iga Karwacka, Julia Ostapowicz, Małgorzata Cisek, Andrzej Adam Mackiewicz, Patrycja Czerwińska

Supplementary materials

1.1 Spearman correlation – Immune score vs. BrD family members' expression

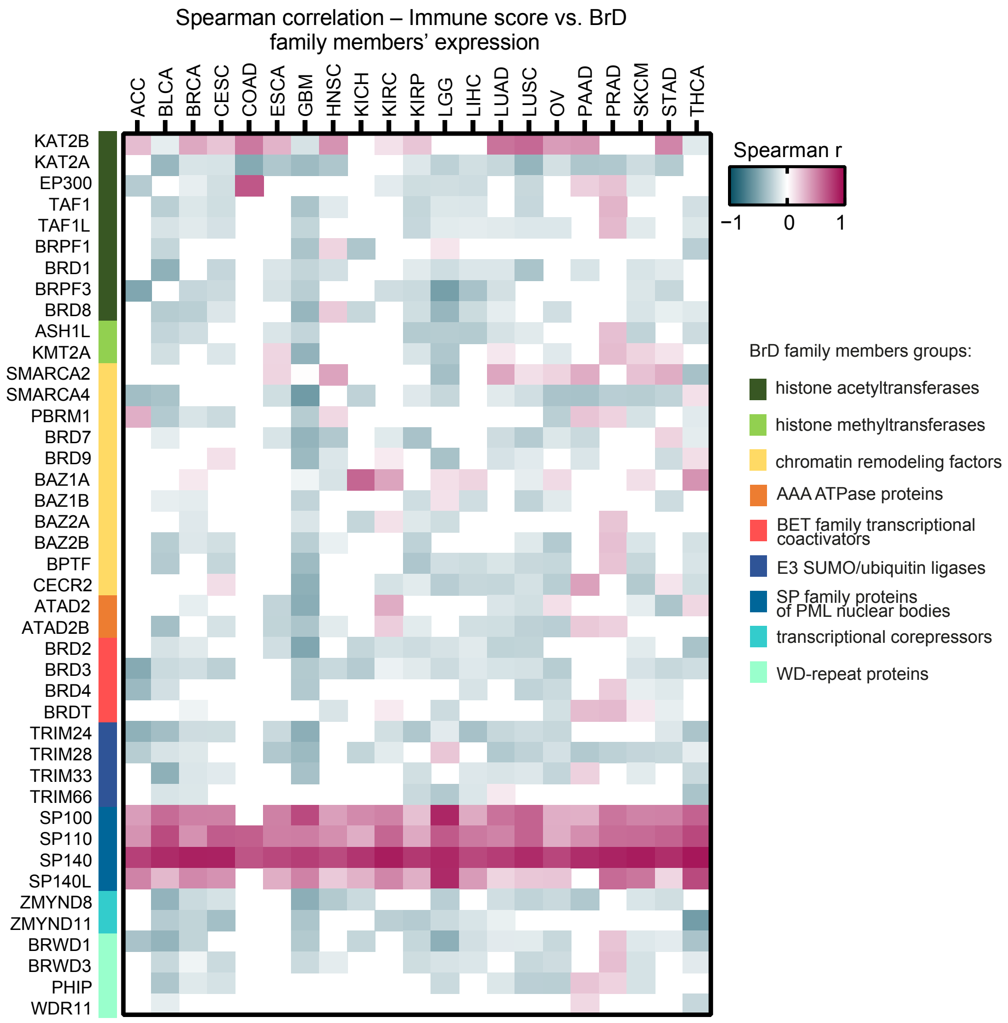

Figure S1. Estimate score signature associated with the expression of BrD family members. The heatmap of Spearman's correlation between the BrD family members expression and the Immune score among 21 solid tumor types in distinct TCGA cohorts. Color on the heatmap denotes either expression is upregulated (pink) or downregulated (dark blue). White squares denote no statistical significance ($p > 0.05$).

1.2 Spearman correlation – Stromal score vs. BrD family members' expression

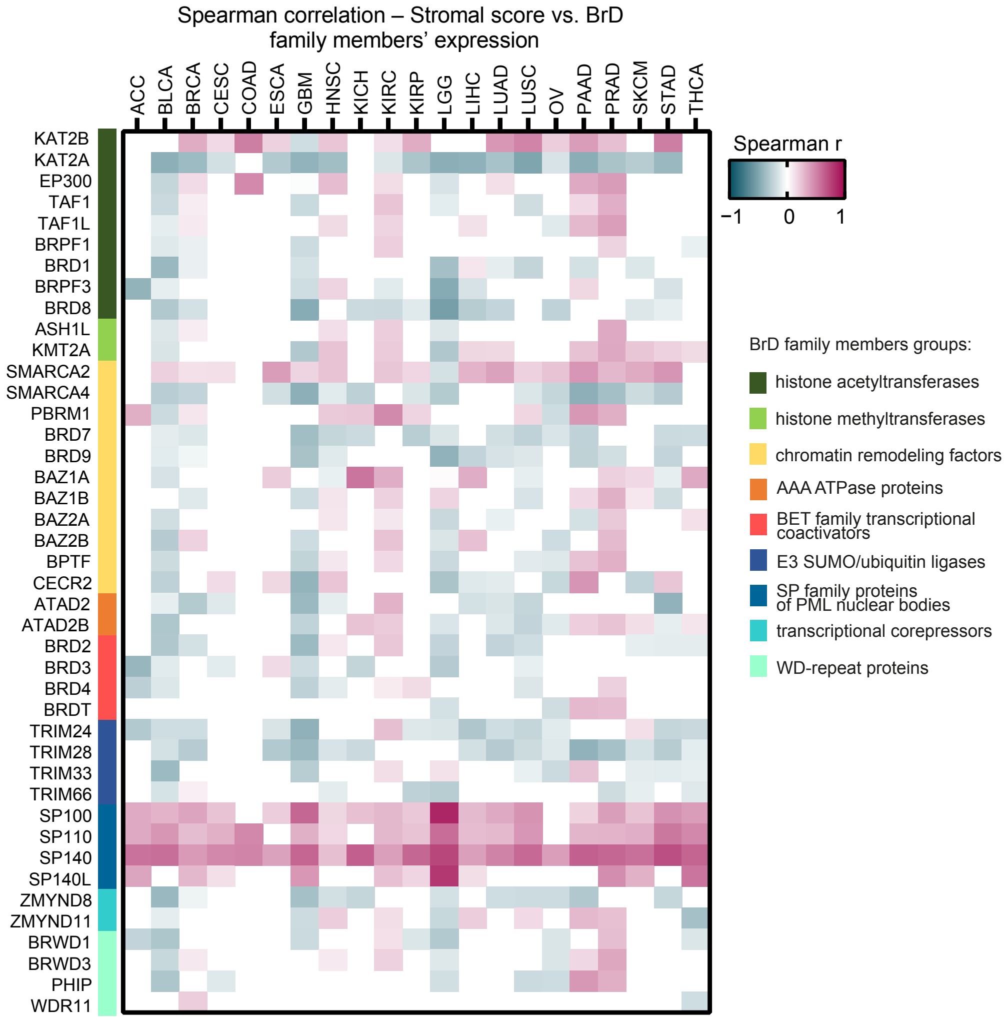

Figure S2. Estimate score signature associated with the expression of BrD family members. The heatmap of Spearman's correlation between the BrD family members expression and the Stromal score among 21 solid tumor types in distinct TCGA cohorts. Color on the heatmap denotes either expression is upregulated (pink) or downregulated (dark blue). White squares denote no statistical significance (p > 0.05).

1.3 The correlation between selected BrD family members' expression and immune cells' infiltration in HNSC and BLCA

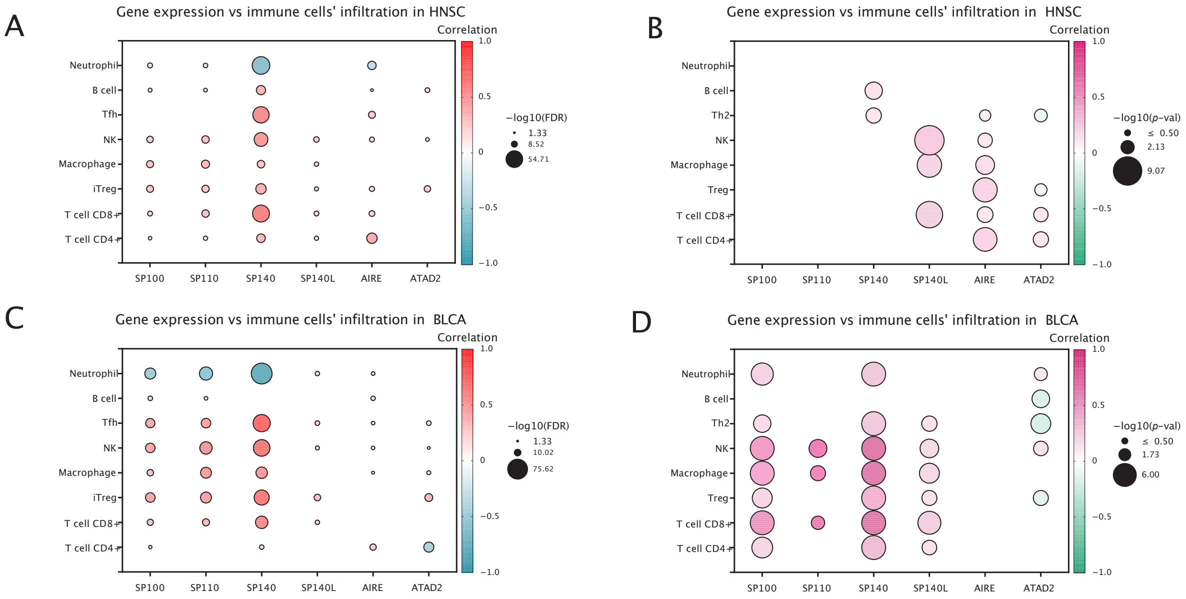

Figure S3. BrD that belong to the SP subfamily are positively associated with tumor infiltration status. The correlation between selected BrD family members' expression and immune cells' infiltration in HNSC (A) and BLCA (C) using the GSCA database [http://bioinfo.life.hust.edu.cn/GSCA/]. Red and blue denote positive and negative correlations, respectively. Bubble size depicts statistical significance (-log10(FDR)). The correlation between selected BrD family members' expression and immune cells' infiltration in HNSC (B) and BLCA (D) using the TIP database [http://biocc.hrbmu.edu.cn/TIP/pancancerAnalysis.jsp]. Pink and green denote positive and negative correlations, respectively. Bubble size depicts statistical significance (-log10(*p*val)).

1.4 Spearman correlation – immune cell's infiltrate vs. SP family and control genes genes mRNA expression

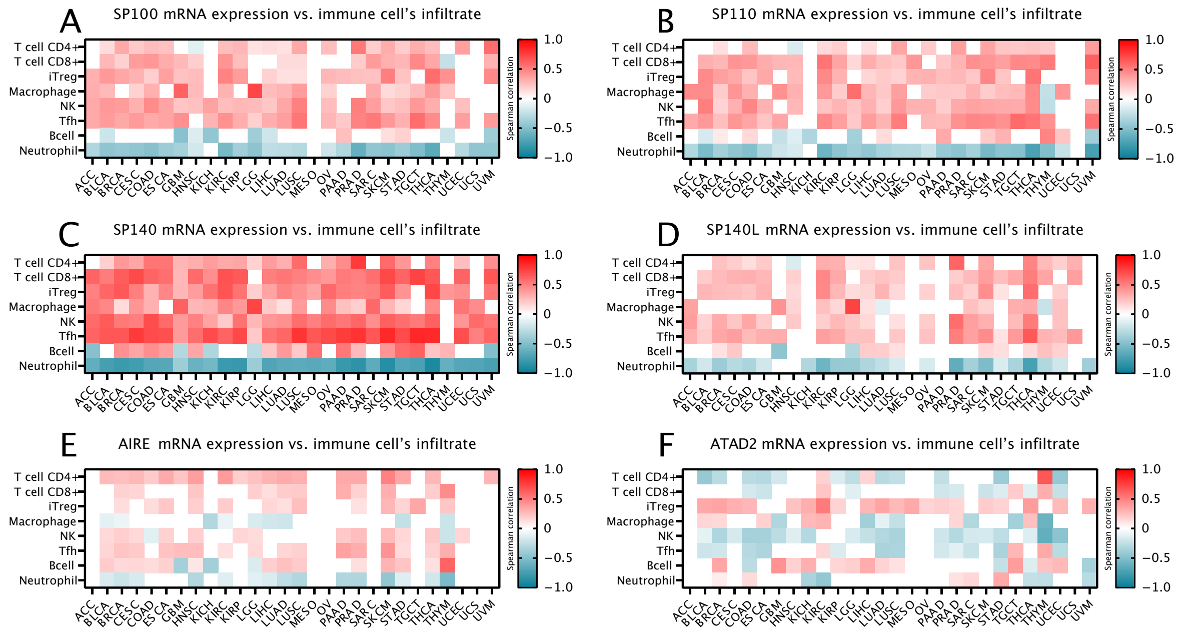

Figure S4. The heatmaps of Spearman's correlation coefficient between SP family genes mRNA expression, namely SP100 (A), SP110 (B), SP140 (C), SP140L (D), also control genes AIRE (E), ATAD2 (F) and specific immune cell's infiltrate - T cell CD4+, T cell CD8+, iTreg, Macrophage, NK cell, Tfh cell, B cell, Neutrophil. Color on the heatmap denotes either correlation is upregulated (red) or downregulated (blue). White squares denote no statistical significance ($p > 0.05$).

1.5 Spearman correlation – populations of immune cells trafficking into tumor (Step IV) vs. SP family and control genes genes expression

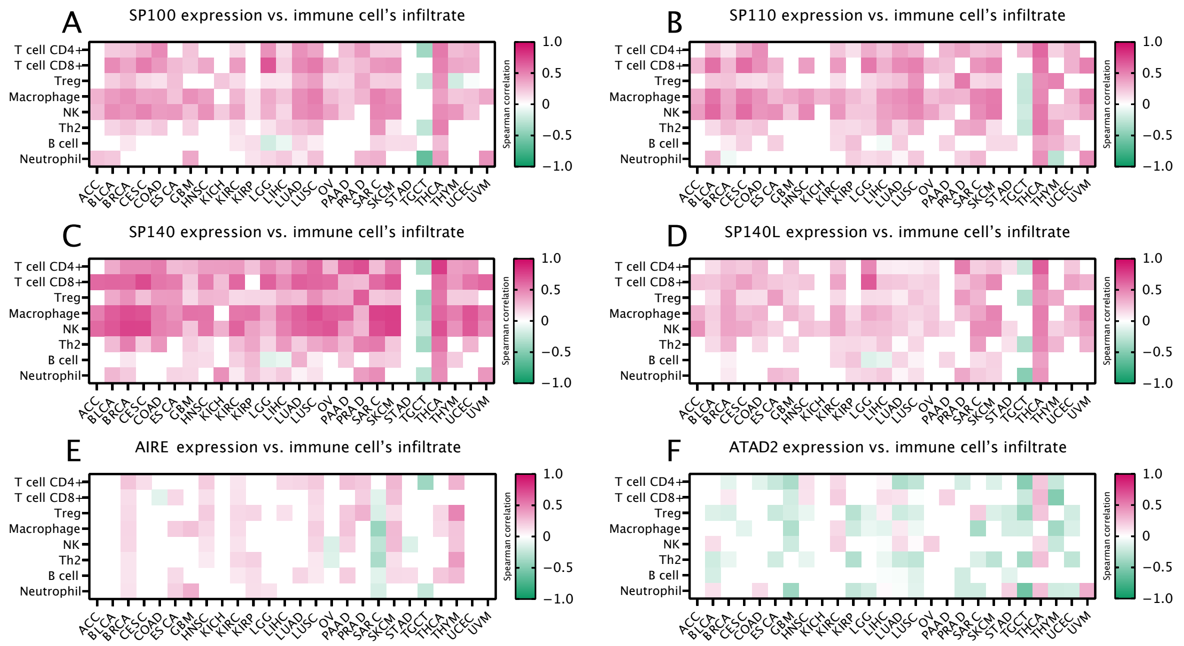

Figure S5. The heatmaps of Spearman's correlation between SP Family genes - SP100 (A), SP110 (B), SP140 (C), SP140L (D), AIRE (E) and ATAD2 (F) expression and the selected populations of immune cells trafficking into tumor (Step IV) from the seven-step cancer-immunity cycle from Tracking Tumor Immunophenotype database (TIP) - T cell CD4+, T cell CD8+, Treg, Macrophage, NK cell, Th2 cell, B cell, Neutrophil. Color on the heatmap denotes either correlation is upregulated (magenta) or downregulated (green). White squares denote no statistical significance ($p > 0.05$).

1.6 Spearman correlation – immune infiltration level of T cell CD8+, T cell CD4+ and Macrophage infiltration vs. SP family and control genes genes expression

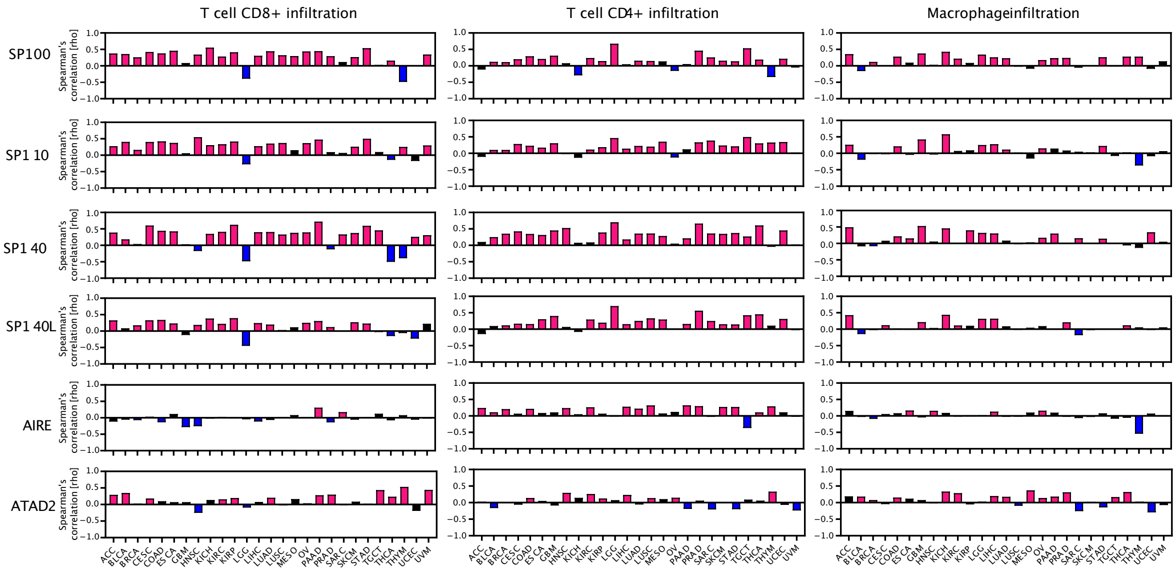

Figure S6. The relationship between infiltrates estimation value and gene expression. The Spearman's correlation between SP family (SP100, SP110, SP140, SP140L) and control (AIRE, ATAD2) genes' expression and immune infiltration level – T cell CD8+; T cell CD4+ and Macrophage infiltration – among 27 solid tumor types. Pink and blue denote positive and negative correlations with statistical significance (p <0.05), respectively, while black denotes no statistical significance (p > 0.05).

1.7 Immune-related Hallmark gene sets for SP family genes

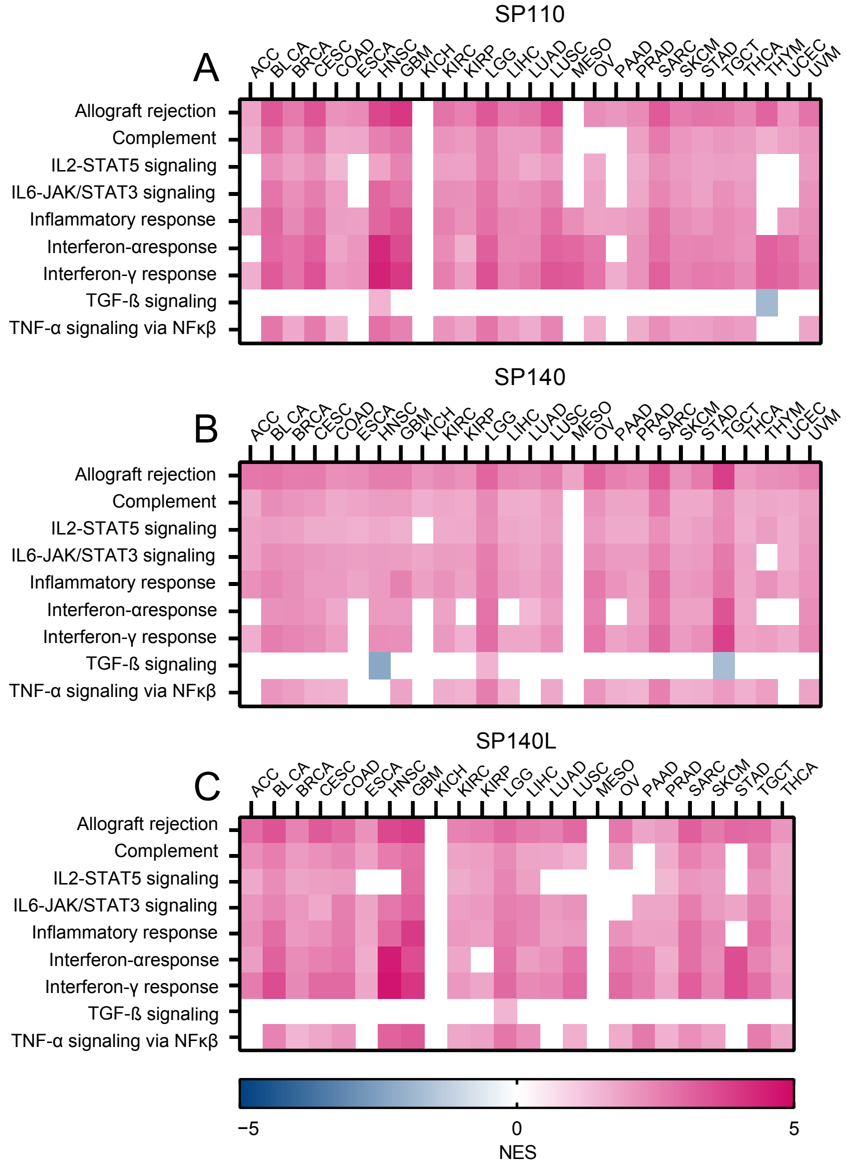

Figure S7. Stemness signature enrichment in the transcriptome profiles associated with the expression of SP family members (*SP100, SP110, SP140, SP140L*) and control genes (*AIRE, ATAD2*). The heatmaps of normalized enrichment scores (NES) of immune-related Hallmark gene sets among 27 solid tumor types for *SP110* (A) and *SP140* (B), or 24 solid tumor types for *SP140L* (C). Color of the heatmap denotes either increase (magenta) or decrease (dark blue) of NES with statistical significance (FDR < 0.05).

1.8 Stemness signature enrichment in the transcriptome profiles associated with the expression of SP family members and control genes.

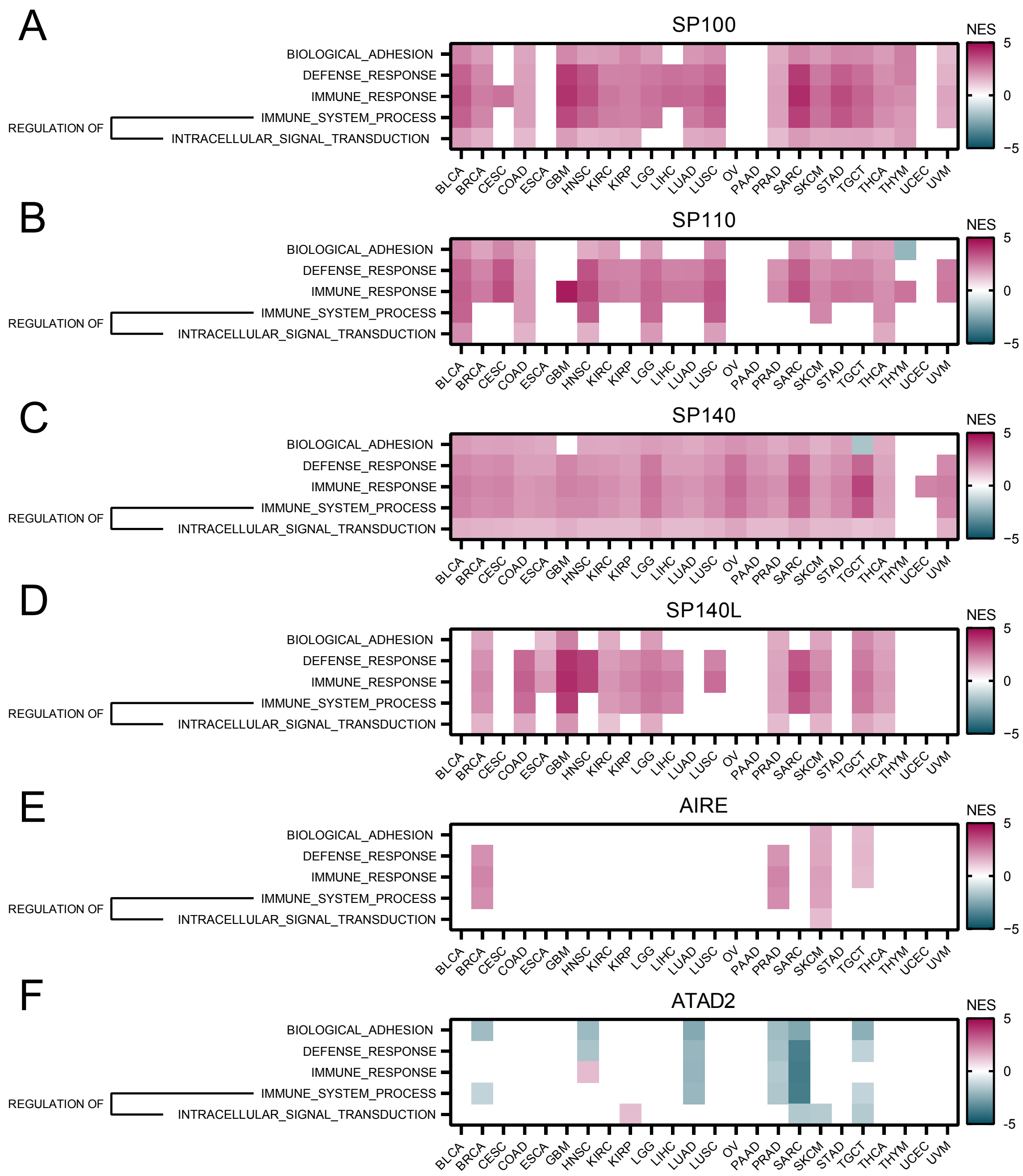

Figure S8. Stemness signature enrichment in the transcriptome profiles associated with the expression of SP family members (*SP100, SP110, SP140, SP140L*) and control genes (*AIRE, ATAD2*). The gene set enrichment analysis (GSEA) was performed using significantly DEGs ($p < 0.05$, FDR < 0.05) in TCGA patients divided into low-expressing or high-expressing BrD cohorts (using the mean expression of each gene as a cut-off). The heatmaps of normalized enrichment scores (NES) of C5.go.bp ontology gene sets among 24 solid tumor types for *SP100* (A), *SP110* (B), *SP140* (C), *SP140L* (D), *AIRE* (E) and *ATAD2* (F). Color of the heatmap denotes either increase (magenta) or decrease (dark blue) of NES with statistical significance (FDR < 0,05).

1.9 The hazard ratio of death for patients with high T cell CD8+, T cell CD4+ or Macrophage infiltration level.

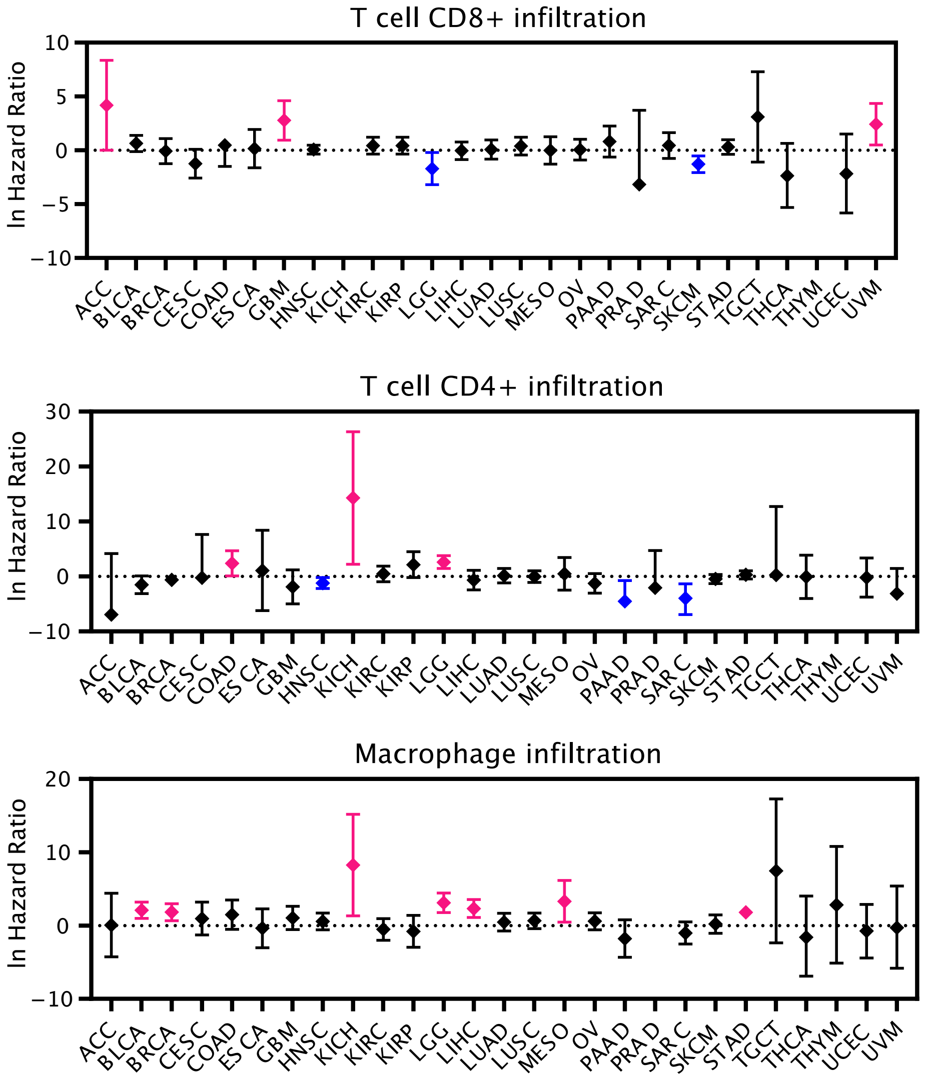

Figure S9. The hazard ratio (lnHR) of death for patients with high T cell CD8+, T cell CD4+ or Macrophage infiltration level. Pink and blue markers indicate statistically significant results with increased or decreased hazard ratios, respectively. Black markers indicate results with no statistical significance ($p > 0.05$). The hazard ratios are given with 95% confidence intervals (CIs).
